# Regenerative Potential of Platelet Concentrate Lysate in Mechanically Injured Cartilage and Matrix-Associated Chondrocyte Implantation In Vitro

**DOI:** 10.3390/ijms222413179

**Published:** 2021-12-07

**Authors:** Jan-Tobias Weitkamp, Bernd Rolauffs, Moritz Feldheim, Andreas Bayer, Sebastian Lippross, Matthias Weuster, Ralf Smeets, Hendrik Naujokat, Alan Jay Grodzinsky, Bodo Kurz, Peter Behrendt

**Affiliations:** 1 Department of Anatomy, Christian-Albrechts-University Kiel, 24118 Kiel, Germany; Dweezil_Feldheim@hotmail.de (M.F.); a.bayer@anat.uni-kiel.de (A.B.); bkurz@anat.uni-kiel.de (B.K.); peter.behrendt@uksh.de (P.B.); 2 Department of Oral and Maxillofacial Surgery, Division of Regenerative Orofacial Medicine, University Medical Center Hamburg-Eppendorf, 20251 Hamburg, Germany; r.smeets@uke.de; 3 Department of Oral and Maxillofacial Surgery, University Medical Center Schleswig-Holstein, Campus Kiel, 24105 Kiel, Germany; hendrik.naujokat@uksh.de; 4 G.E.R.N. Research Center for Tissue Replacement, Regeneration & Neogenesis, Department of Orthopedics and Trauma Surgery, Faculty of Medicine, Medical Center—Albert-Ludwigs-University of Freiburg, 79085 Freiburg im Breisgau, Germany; bernd.rolauffs@uniklinik-freiburg.de; 5 Clinic for Orthopedic and Trauma Surgery, University Medical Center Schleswig-Holstein, Campus Kiel, 24015 Kiel, Germany; sebastian.lippross@uksh.de; 6 Clinic for Trauma Surgery, Diako Hospital Flensburg, 24939 Flensburg, Germany; weuster@diako.de; 7 Department of Oral and Maxillofacial Surgery, University Medical Center Hamburg-Eppendorf, 20251 Hamburg, Germany; 8 Department of Biological Engineering, Massachusetts Institute of Technology, Cambridge, MA 02142, USA; alg@mit.edu; 9 Department of Electrical Engineering and Computer Science, Massachusetts Institute of Technology, Cambridge, MA 02142, USA; 10 Department of Mechanical Engineering, Massachusetts Institute of Technology, Cambridge, MA 02142, USA

**Keywords:** cartilage, platelet concentrate, injury, osteoarthritis, ACI, IL-10

## Abstract

Adjuvant therapy in autologous chondrocyte implantation (ACI) can control the post-traumatic environment and guide graft maturation to support cartilage repair. To investigate both aspects, we examined potential chondro-regenerative effects of lysed platelet concentrate (PC) and supplementary interleukin 10 (IL-10) on mechanically injured cartilage and on clinically used ACI scaffolds. ACI remnants and human cartilage explants, which were applied to an uniaxial unconfined compression as injury model, were treated with human IL-10 and/or PC from thrombocyte concentrates. We analyzed nuclear blebbing/TUNEL, sGAG content, immunohistochemistry, and the expression of COL1A1, COL2A1, COL10A1, SOX9, and ACAN. Post-injuriously, PC was associated with less cell death, increased COL2A1 expression, and decreased COL10A1 expression and, interestingly, the combination with Il-10 or Il-10 alone had no additional effects, except on COL10A1, which was most effectively decreased by the combination of PC and Il-10. The expression of COL2A1 or SOX9 was statistically not modulated by these substances. In contrast, in chondrocytes in ACI grafts the combination of PC and IL-10 had the most pronounced effects on all parameters except ACAN. Thus, using adjuvants such as PC and IL-10, preferably in combination, is a promising strategy for enhancing repair and graft maturation of autologous transplanted chondrocytes after cartilage injury.

## 1. Introduction

Focal cartilage lesions in young and active patients predispose to degenerative joint disorders [1]. Although lager size defects can be treated by using tissue engineered ACI products, there is evidence that persistent inflammation and induction of catabolic factors lead to matrix degradation and cell death, which results in progressive joint degeneration [2]. Proinflammatory cytokines, aggrecanases, and collagen cleavage fragments et cetera can be measured in the synovial fluid and serum in elevated concentration up to 2 years after trauma and similar mechanisms are presumable for cartilage injuries [3]. A missing key in the successful repair of those injuries may be the treatment of this post-injurious joint milieu. In this context, it is important that the treatment agent not only regulates the immune response, but also do not harm the graft maturation of a cartilage regenerative intervention. A combinatory approach of tissue engineered reconstruction together with the application of chondro-protective biological factors may antagonize joint degeneration in its beginning and additionally supports graft maturation.

In this context, autologously obtained platelet-rich plasma (PRP) has been used in many areas of regenerative medicine since its introduction in the late eighties of the last century [4]. The coagulation and growth factors contained in blood plasma are intended to speed up physiological healing processes and improve tissue regeneration and, thus, the latter has been introduced as a suitable culture supplement for tenocytes, keratocytes and fibroblasts [5,6]. In patients suffering from osteoarthritis (OA) intraarticular injections of PRP can ameliorate pain and increase function [7,8]. However, certain blood plasma components might not be favorable for chondrogenic tissue regeneration as they stimulate bone matrix formation and collagen type I expression. For example, increased type 1 collagen expression induced by PRP has been demonstrated recently in chondrocytes [9]. Therefore, it remains questionable whether PRP is appropriate for cartilage regeneration patients with early OA. In that regard, a connatural plasma-free lysate of concentrated platelets (PC) has been introduced, which is produced solely from platelets concentrate and consists of concentrated alpha granules of activated blood platelets [10]. PC used in this study has often been introduced as “PRGF” (platelet released growth factors), which certainly is misleading with respect to the terminology introduced by [11], who named the same constitute plasma rich in growth factors. The last mentioned PRGF is a platelet-enriched plasma. Therefore, it has correctly been criticized, the “P” makes the difference in PRGF [4], which is why we called platelet concentrate lysate in this study “PC” according to terminology introduced by Gulliksson et al. [12]. PC increases the platelet density by 10 times and does not contain plasma-derived proteins and growth factors. It has been far less investigated, and reports on potential chondro-protective and chondro-permissive effects remain so far unavailable.

A major drawback of PRP and potentially PC is its heterogenous mixture of various mediators, which can lead to undesired effects like hypertrophy and inflammation. Factors like interleukin 1𝛽 and insulin-like growth factor 1 (IGF-1) are contained in PRP in a considerable high concentration. Therefore, the addition of an immunomodulating mediator like interleukin 10 (IL-10) might be a potential strategy to control these side-effects of PRP and its equivalents like PC. IL-10 has already been demonstrated to protect post-traumatic cartilage degeneration and to support chondrogenic graft maturation in collagen-based scaffolds for autologous chondrocyte implantation [13,14].

Based on the suggested combinatory adjuvant approach, the aim of this study was to investigate the influence of PC on post-injurious cartilage microenvironment and whether it simultaneously promotes chondrogenesis in a clinically established type 1/3 collagen-based scaffold for ACI treatment. Biological effects on injured cartilage were assessed using a well-established in vitro injury model and IL-10 as post-injurious immunomodulating substance [13,14,15,16,17,18,19,20,21].

## 2. Results

### 2.1. Apoptosis in Mechanically Injured Cartilage

**Figure 1:**
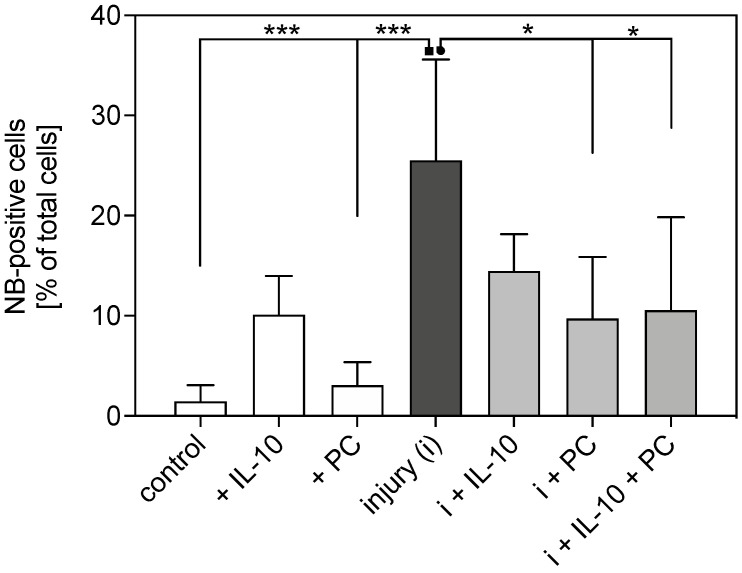
Anti-apoptotic effects of IL-10 and platelet concentrate (PC) in mechanically injured cartilage. Nuclear blebbing (NB)-positive of explants cultured for 4 days in free-swelling conditions and after mechanical injury with and w/o treatment of IL-10 and PC. Asterisks indicate significant differences with * *p* < 0.05 *** *p* < 0.001. ▪ dot indicates experimental group to which the others are statistically significantly different. Data are presented as mean + SD (*n* = 6).

The apoptotic cell rate was analyzed 4 days after cartilage injury and compared to samples in free-swelling conditions. Nuclear blebbing revealed that mechanical injurious compression significantly induced cell death 16-fold (*p* < 0.001) which was significantly reduced by PC (−61%) supplementation and co-treatment (−60%) with PC and IL-10 (injury vs. injury + PC: *p* = 0.0305; injury vs. injury + PC + IL-10: *p* = 0.0483; Figure 1).

### 2.2. Alterations of the Chondrogenic Phenotype in Mechanically Injured Chondrocytes

In injured human cartilage explants the supplementation of PC and co-treatment with IL-10 and PC significantly enhanced mRNA expression of chondrogenic markers (COL2A1, ACAN) compared to untreated injured samples (mRNA expression of COL2A1 in experimental groups with injury + PC vs. injury: *p* = 0.0087; injury + IL-10 + PC vs. injury: *p* = 0.0251; mRNA expression of ACAN in experimental groups with injury + PC vs. injury: *p* = 0.0094; Table 1). Samples cultured with sole IL-10 or PC application had a mild increase in COL2A1, SOX9 and ACAN mRNA expression (Table 1). In particular, transcription levels of SOX9 significantly increased under sole PC treatment compared to samples with mechanical injury (*p* = 0.0097; Table 1). Markers of cell dedifferentiation (COL1A1, COL10A1) were significantly induced after injury. Co-treatment with IL-10 and PC indicated a reduced post-injurious COL1A1 mRNA expression in human cartilage explants without reaching statistical significance (Table 1). Cell hypertrophy marker COL10A1 was significantly reduced in all experimental groups compared to injured ones (*p* < 0.0001; Table 1). Further, the COL2A1/COL1A1 ratio indicated chondroprotective effects of PC and IL-10. In this context, the COL2A1/COL1A1 ratio presents a balance between a functional chondrocyte phenotype, as found in intact cartilage, and a modulated proliferative in vitro phenotype and values > 1 indicate a functional phenotype comparable to intact cartilage [22,23,24]. To summarize, supplementation of IL-10 and PC led to stabilization of the chondrogenic phenotype in mechanically injured human cartilage explants. Most significant rescue effects were observed for hypertrophy marker COL10A1 when compared to untreated injured cartilage (IL-10 −72% COL10A1 expression; PC −67% COL10A1 expression; −82% COL10A1 expression).

### 2.3. Post-Injurious Changes of Extracellular Matrix Compounds in Human Cartilage Explants

**Figure 2:**
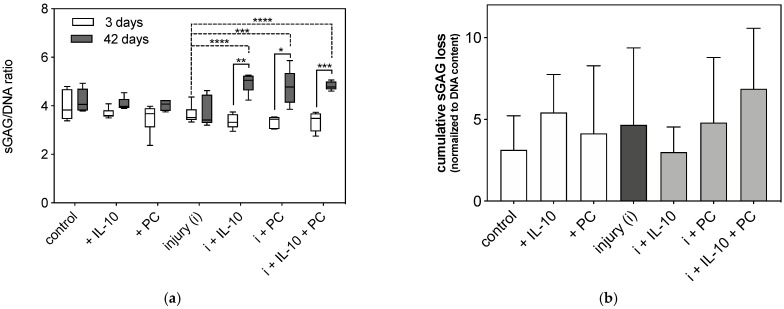
Sulphated glycosaminoglycan (sGAG) content and release in post-injurious (injury, i) human cartilage explants. Total sGAG content (**a**) and cumulative sGAG release (**b**) were analyzed by 1,9-dimethylmethylene blue (DMMB) assay and normalized to corresponding sample DNA content (sGAG/DNA ratio). sGAG content was detected after 3 and 42 days (**a**), sGAG release was measured over 42 days cultivation time (**b**). Asterisks indicate statistically significant differences with * *p* < 0.05, ** *p* < 0.01 *** *p* < 0.001 and **** *p* < 0.0001. Data are presented as mean ± SD (A: box whiskers plots), (*n* = 6).

Changes in the amount of ECM compounds were detected by DMMB assay as well as histology studies using toluidine blue staining and type 2 and type 1 collagen immunohistochemistry. Firstly, sGAG loss of human cartilage explants was detected after 3 and 42 days using a DMMB assay and then normalized to the DNA content of the corresponding samples. In addition, cumulative sGAG loss into the cell culture supernatant was measured over 6 weeks and normalized to the DNA content of corresponding samples at day 42. The cumulative sGAG loss of cartilage explants did not differ significantly in groups without mechanical injury (Figure 2). Injured samples at day 42 had the lowest sGAG content compared to all other groups. Supplementation of IL-10 and PC to the chondropermissive culture medium significantly increased sGAG content after 42 days compared to injured groups after 3 days (injury day 3 vs. injury + IL-10 day 42: *p* < 0.0001; injury day 3 vs. injury + PC day 42: *p* = 0.0002, injury day 3 vs. injury + IL-10 + PC day 42: *p* < 0.0001; Figure 2a). Also, during culture the presence of adjuvant IL-10 and PC also significantly increased sGAG content in injured samples (injury + IL-10 day 3 vs. injury + IL-10 day 42: *p* = 0.0051; injury + PC day 3 vs. injury + PC day 42: *p* = 0.0238; injury + IL-10 + PC day 3 vs. injury + IL-10 + PC day 42: *p* = 0.0002) indicating an induction of sGAG neosynthesis (Figure 2a). In addition, higher sGAG content was paralleled by increased cumulative sGAG loss into the cell culture supernatant in groups treated with IL-10 and with PC (Figure 2b).

**Figure 3:**
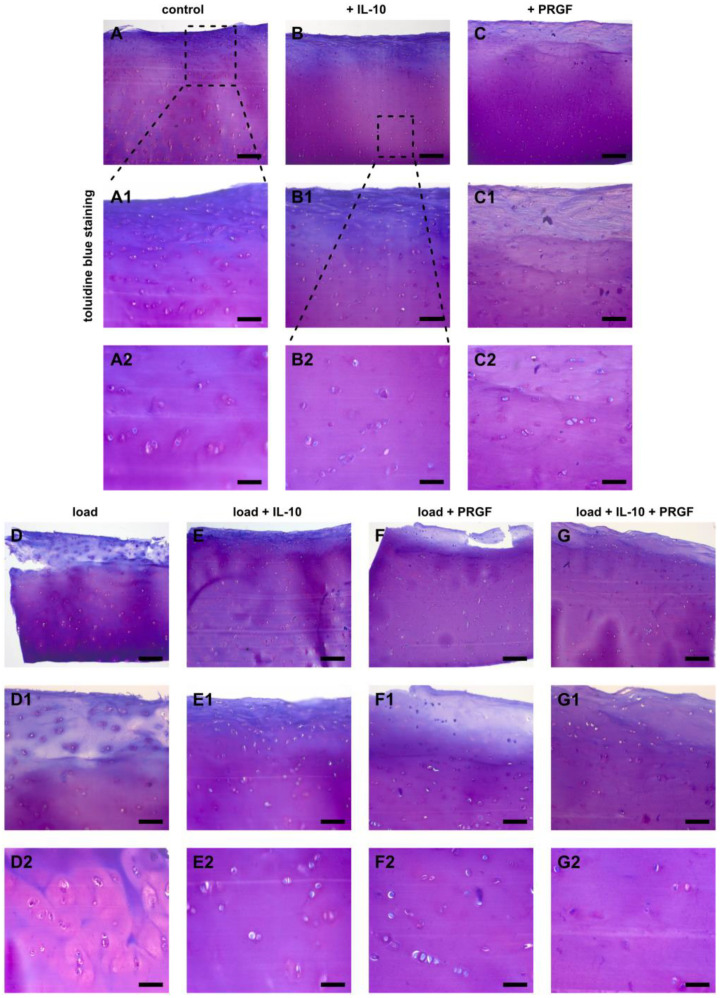
Changes of post-injurious human cartilage with treatment of IL-10 and PC. Toluidine blue staining of humane cartilage explants treated with IL-10 and PC with and without mechanical injury. Explants were cultured for 42 days after injury. (**A**–**G**) overview, (**A1**–**G1**) magnification of the superficial zone, (**A2**–**G2**) magnification of the deep layer. Bar 200 μm (A1/A2–G1/G2 100 µm).

This quantitative analysis was accompanied by histology studies with toluidine blue. After 42 days, injured samples that were treated with IL-10 showed enhanced metachromasia in superficial and deeper hyaline cartilage layers similar to untreated and non-injured explants (Figure 3B,E). Injured samples showed a significant reduction in sGAGs in the superficial layers, which was mitigated by IL-10 application (Figure 3D,E). Sole PC did not prevent sGAG loss in the superficial layers of injured cartilage explants like IL-10 (Figure 3F). However, the co-treatment again resulted in enhanced metachromasia in the superficial and deep layers after mechanical injury (Figure 3G).

**Figure 4:**
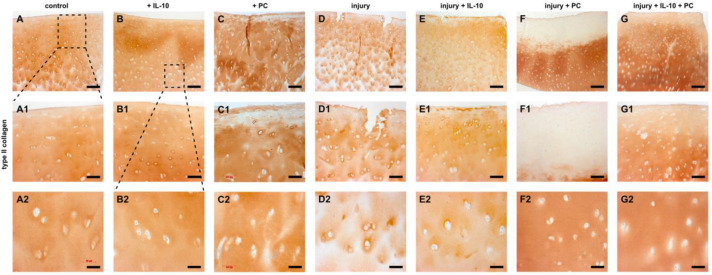
Immunohistochemistry of type 2 collagen in post-injurious human cartilage explants. Immunohistochemistry of type 2 collagen of humane cartilage explants treated with IL-10 and PC with and without mechanical injury. Explants were cultured for 42 days after injury. (**A**–**G**) overview, (**A1**–**G1**) magnification of the superficial zone, (**A2**–**G2**) magnification of the deep layer. Bar 200 μm (A1/A2–G1/G2 100 µm).

**Figure 5:**
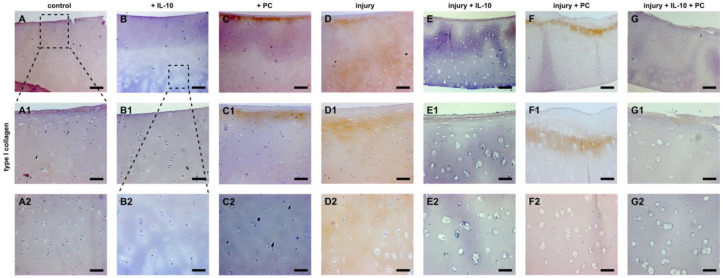
Immunohistochemistry of type 1 collagen accumulation in post-injurious human cartilage explants. Immunohistochemistry of type 1 collagen of humane cartilage explants treated with IL-10 and PC with and without mechanical injury. Explants were cultured for 42 days after injury. (**A**–**G**) overview, (**A1**–**G1**) magnification of the superficial zone, (**A2**–**G2**) magnification of the deep layer. Bar 200 μm (A1/A2–G1/G2 100 µm).

Immunohistochemistry of type 2 and type 1 collagen revealed a similar pattern. Non-injured, IL-10 treated cartilage explants displayed enhanced type 2 collagen content compared to control groups. Application of PC resulted in type 2 collagen loss in the superficial layers (Figure 4C). Injured samples revealed a distinct loss especially in the superficial zone and the interterritorial matrix (Figure 4E). This effect was distinctly mitigated by IL-10 in superficial layers and PC in deeper layers (Figure 4F,G). PC strongly enhanced type 2 collagen accumulation in the interterritorial matrix. The co-treatment resulted in prevention of type 2 collagen loss through all depths. Immunoprecipitation of type 1 collagen confirmed the previously observed results. Once again, addition of PC to the culture medium resulted in changes in the superficial layers of cartilage explants with and without mechanical injury by enhanced accumulation of type 1 collagen, which was not observed in IL-10 treated groups (Figure 5C,F). Mechanical injury led to a strong type 1 collagen deposition through all depths of hyaline cartilage explants (Figure 5D). The presence of the adjuvant IL-10 significantly reduced type 1 collagen accumulation in injured and PC treated samples (Figure 5E,G).

To summarize, the presence of IL-10 showed greatest chondroprotective effects in terms of quantitative sGAG retention in human cartilage explants after mechanical injury. Histology studies showed that IL-10 strongly enhanced ECM integrity and mitigated potentially negative effects of PC.

### 2.4. Chondrogenic Potential of IL-10 and PC Treatment in Cellularized ACI Grafts

The chondrogenic potential of IL-10 and PC treatment in cellularized ACI grafts was analyzed by qPCR, DMMB assay as well as histology studies.

Co-treatment with IL-10 and PC displayed a significant chondrogenic potential in chondrocytes embedded in a type 1/3 collagen ACI graft. Cell differentiation was significantly enhanced, which was demonstrated by altered mRNA expression of COL2A1, COL1A1, and COL2A1/COL1A1 ratio (Table 2). Transcription levels of COL2A1 mRNA revealed a significant increase in experimental groups under co-treatment compared to sole application with of IL-10 and PC, respectively, after 28 days (+IL-10 vs. +IL-10 + PC: *p* = 0.0068; +PC vs. +IL-10 + PC: *p* = 0.0315; Table 2). On the other hand, COL1A1 mRNA expression was significantly reduced in groups with co-treatment compared to sole PC (*p* = 0.0055; Table 2). COL2A1/COL1A1 ratio paralleled these results indicating chondrogenic cell differentiation. Gene expression levels of ACAN showed no significant changes, but cell de-differentiation marker COL10A1 demonstrated a tendency for less cell hypertrophy in groups supplemented with PC and IL-10 (Table 2).

**Figure 6:**
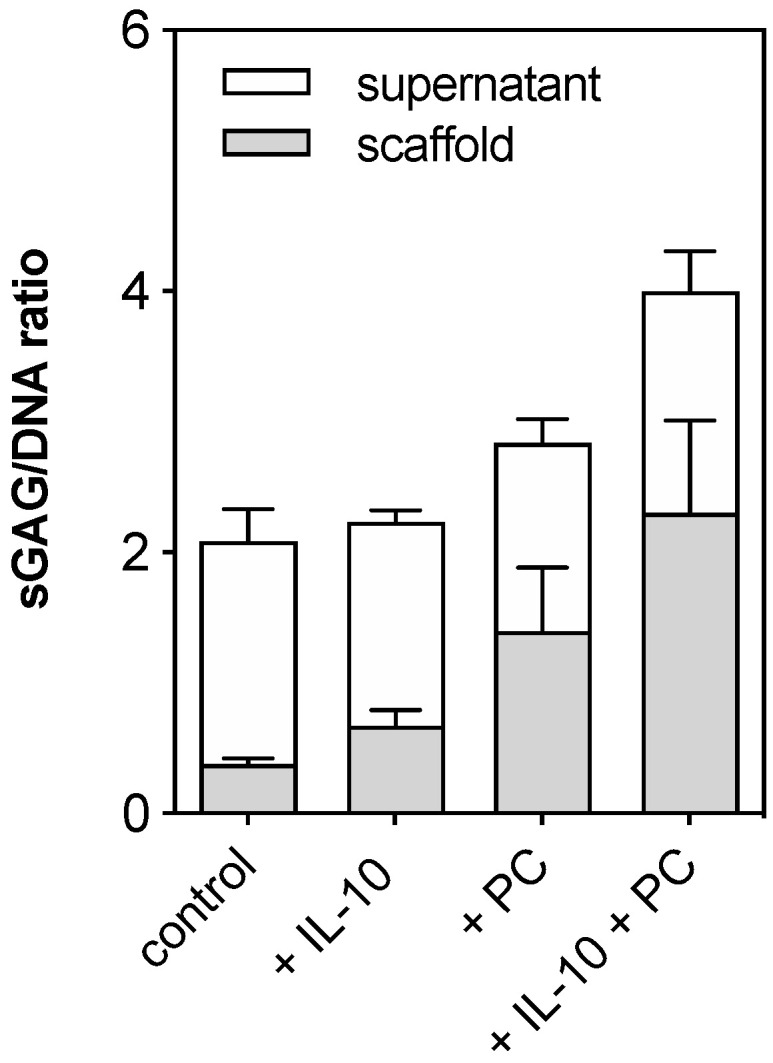
Quantification of sGAG biosynthesis in ACI grafts and the culture supernatant. sGAG content and release normalized to the DNA content in ACT grafts after 28 days of culture in chondropermissive medium and additional treatment of IL-10 and PC. Data are presented as mean + SD (*n* = 3).

**Figure 7:**
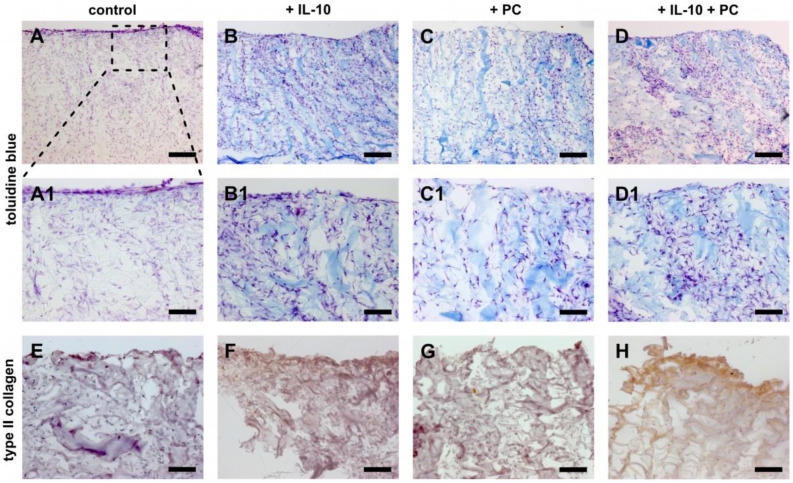
Histological analysis of ACI grafts treated with IL-10 and PC. (**A**–**D**) Toluidine blue staining of cellularized type 1/3 collagen matrix cultivated for 28 days in chondropermissive medium with additional treatment of IL-10 and PC as well as co-treatment. Representative images of each treatment group after 28 days of culture. (**E**–**H**) Immunohistochemical staining of collagen type 2 in cellularized type 1/3 collagen matrix. Images were taken of the macroporous layer. Bar 200 µm (**E**–**H**,**A1**–**D1** 100 μm).

Furthermore, quantification of sGAG biosynthesis normalized to DNA content showed a tendency for enhanced chondrogenic ACI graft maturation under co-treatment of both supplements (Figure 6). This was paralleled by ECM neosynthesis observed by toluidine blue staining and immunohistochemistry of type 2 collagen. The presence of sole IL-10 and sole PC led to enhanced metachromasia (purple staining due to negative charges), which was stronger in samples under co-treatment (Figure 7A–D). Type 2 collagen immunoprecipitation was only observed in the same treatment group at the cell-seeding side of the graft (Figure 7H). Application of one factor alone did not result in increased staining (Figure 7F,G). Type 1 collagen immunohistochemistry is not shown because ACI grafts consist of type 1/3 collagen and therefore overlay all potential effects.

## 3. Discussion

Successful treatment of larger size traumatic cartilage injuries potentially requires control of the post-traumatic joint environment and filling of the cartilage defect by using a tissue engineered construct like ACI. The first has not been introduced in the clinical application yet. Therefore, we aimed to investigate potential adjuvant agents and revealed that a combinatory approach of PC and IL-10 can not only ameliorate certain mechanisms of post-injurious cartilage degeneration in vitro, but also enhanced the expression of cartilage-like markers indicating improved ACI graft maturation. Single therapy of PC was less effective indicating that the regenerative potential of PC needs guidance by an immunomodulating mediator like IL-10 to specifically aim cartilage-like tissue regeneration.

In detail, the application of PC alone after injury to cartilage explants was associated with less cell death, increased COL2A1 expression, and decreased COL10A1 expression. Although the combination of PC with Il-10 or Il-10 alone had no additional effects on cell death or on COL2A1, it was the combination of PC and Il-10 that was most effectively in decreasing COL10A1 expression. Thus, in injured cartilage explants, chondrogenic effects were achieved with PC alone but anti-hypertrophic effects were achieved with both PC and Il-10. Relevant was also that the expression of COL1A2 (or SOX9) within injured cartilage explants were not modulated by these substances but this might be a statistical effect and subsequent studies will need to confirm this point. Also highly interesting was the fact that in chondrocytes in ACI grafts the combination of PC and IL-10 had the most pronounced effects on COL1A2 and COL2A1 expression and the COL2A1/COL1A2 ratio, whereas ACAN and SOX9 expression remained statistically largely unaffected. Thus, targeting post-injurious cartilage tissue and ACI chondrocytes simultaneously, e.g., when using ACI post-traumatically, would benefit from a combination of PC and IL-10, whereas targeting cell death as well as modulating COL1A2 and COL2A1 expression in the tissue would perhaps require only PC.

In our study, PC treatment of OA chondrocytes as well as injured OA cartilage stabilized the chondrogenic phenotype. Additionally, PC and IL-10 reduced post-injurious type 1 and 10 collagen expression, with the combinatory treatment being more effective than the single application. Hereby, a bio-effective dosage of 100 pg/mL IL-10 was used as reported before [14]. In fact, part of PC effect might be due to IL-10 induction, which has been demonstrated for PRP treatment of chondrocytes and synoviocytes [25,26]. However, these findings were partially in contrast with the histological analysis, in which IL-10 showed distinct ECM preserving effects after cartilage injury while PC was less protective. PC did not recover type 2 collagen loss in the superficial third of the explants, while IL-10 seemed to be much more potent in preserving ECM integrity. Also, PC treatment increased type 1 collagen deposition, which may be indicative for fibrocartilage regeneration. Strong induction of type 1 collagen by PC has been recently demonstrated in a transcriptome analysis of keratinocytes [27] and may be due to various factors in platelets alpha granula.

A similar pattern was observed for GAGs. As reported previously, sGAG loss decreased from the cartilage surface to deeper layers, which is likely due to a decreasing strain throughout the thickness of the explant. PC was less effective in preserving sGAG loss in the superficial zone compared to post-injurious IL-10 and the combinatory treatment. In this regard, Patwari et al. demonstrated that proinflammatory cytokines cause a synergistic loss of proteoglycans from mechanically injured cartilage [28]. It is hypothesized that IL-10 may better control these degenerative pathways as PC does. Co-treatment of IL-10 and PC abolished the observed drawbacks of PC in the histological analysis, which strongly promotes the combined application.

Similar effects were seen in PC treated ACI remnants, in which the combinatory treatment with PC and IL-10 was most effective in enhancing cartilaginous marker expression. PC induced type 1 and 10 collagen mRNA expression, which was reduced by co-treatment with IL-10. It has to be taken into account that according to the manufacturers protocol chondrocyte redifferentiation following monolayer cell expansion was already initiated prior to implantation. Similar effects were seen for sGAG neosynthesis and IHC staining of collagen. Given the heterogeneity of platelets alpha granula it does not seem surprising that PC as well as PRGF also induce fibrocartilage and cell hypertrophy. In this regard the application of different immuno-modulating agents similar to IL-10 have been investigated to eradicate undesired hypertrophic and fibrocartilage-like responses [29].

Although the in vitro design of this study limits its clinical translation, there is growing evidence to support an adjuvant therapy in cartilage regeneration [30]. Distinct changes within the synovial fluid have been revealed following joint injury and few biomarkers like matrix metalloproteases (MMPs) have been correlated to clinical ACI failure [31]. In addition to the data presented in this study, it has previously been reported by our study group that IL-10 reduces post-traumatic MMP increase in cartilage tissue. Therefore, utilizing advantages of the regenerative and anti-apoptotic potential of PC and co-treatment with additional cofactors like IL-10 might be particularly effective in the treatment of post-traumatic cartilage defects. However, there is no specific clinical translation of this concept, yet. A related strategy targeting immunomodulation has already been introduced with the application of allogenic or adipose-derived stem cells in cartilage defects and OA [32,33]. The rationale for this is also a locoregional induction of growth factors and cytokines by secretion of those stem cells, which also have an anti-inflammatory and regeneration-promoting effect [34].

Therefore, utilizing advantages of the regenerative and anti-apoptotic potential of PC and co-treatment with additional cofactors like IL-10 may be necessary in order to guide a chondrogenic regenerative pathway and block fibrocartilage formation. Treatment of the post-injurious microenvironment without negatively impacting the ACI construct would be of tremendous importance and remains a missing keystone in successful cartilage treatment. Thus, PC application and IL-10 functionalization of ACI grafts at the time of ACI surgery could be a promising strategy to acquire its beneficial effects locally at the defect site.

## 4. Materials and Methods

### 4.1. Preparation of PC

PC used in this study was prepared from supernatants of freshly donated thrombocyte concentrates (Institute of Transfusion Medicine, University of Schleswig-Holstein, Campus Kiel) derived from leucocyte-depleted haemapheresis according to the officially recommended practice (Richtlinien zur Gewinnung von Blut und Blutbestandteilen und zur Anwendung von Blutprodukten (Hämotherapie), Transfusionsgesetz, Bundesärztekammer, 2010). Thrombocyte concentrates were centrifuged for 10 min at 2000× *g*, washed twice with a sodium citrate buffer (0.11 mM, pH 5.5, 37 °C) and centrifuged again for 10 min at 2000× *g*. Resuspended thrombocytes were stored on ice, lysed by ultrasound and stored at −80 °C for 24 h. Subsequently, the suspension was thawed, the ultrasound procedure was repeated, and the suspension was stored again at −80 °C for 24 h. After thawing, the suspension was centrifuged for 1 min at 18,000× *g*. The supernatant, which is the PC, was stored in aliquots at −20 °C. Thrombocyte concentration exceeded 2–4 × 10^11^ per concentrate (200–450 mL), including less than 5 × 10^5^ leucocytes. Preparation of PC was done as described before [35]. PC used for all experiments was taken from a previously pooled stock (*n* = 5; 38 ± 8.2 years).

### 4.2. Isolation of Articular Cartilage Explants and Preparation of Human Chondrocyte-Laden Collagen Scaffolds

Articular cartilage explants were isolated from the postoperatively remaining femoral heads of patients undergoing joint replacement surgery (*n* = 6; age 55 ± 6.7 years). Therefore, the harvest was organized upon consultation with the orthopedic surgeon, who performed the procedure. After careful opening and partial resection of the joint capsule and osteotomy of the femoral neck, the femoral head can be removed by pulling forces. Thereby only minimal compressive forces are generated. Cartilage for our experiments were harvested from the more peripheral areas of the femoral head that were not macroscopically affected by OA or macroscopically injured by the harvesting procedure. In fact, no suchlike acute injuries were seen in our specimens. Cartilage explants were then prepared as previously described by our study group [21]. Cell-laden ACI grafts (CS; *n* = 3; mean age 32.4 ± 10.7) were obtained from post-operatively remaining type 1/3 collagen scaffolds of patients undergoing matrix-associated ACI treatment (Novocart 3D^®^, TETEC, Reutlingen, Germany). All waste materials used have a patient’s consent and have been approved by the Ethics Committee of Kiel University Hospital (ethic authorization number: D489/15). CS graft material was cut into 5 × 5 mm^2^ samples. CS has a bilayered architecture with a macroporous part (cell seeding side) and a dense side facing the articular joint compartment. After isolation and preparation, explants and graft samples were cultured separately in 96-well plates with 250 µL medium and equilibrated for 24 h at 37 °C in an atmosphere of 5% CO_2_ in serum-free chondropermissive medium: High-glucose Dulbecco’s modified Eagle’s medium (Biochrom, Berlin, Germany) supplemented with 10 mM HEPES buffer (Biochrom, Berlin, Germany), 1 mM sodium pyruvate (PAA Laboratories, Pasching, Germany), 0.1 mM nonessential amino acids (Sigma–Aldrich, Darmstadt, Germany), 0.4 mM proline (Sigma–Aldrich, Darmstadt, Germany), 1% ITS Liquid media supplement (Sigma–Aldrich, Darmstadt, Germany), 100 units/mL of penicillin G and streptomycin (PAA Laboratories, Pasching, Germany).

### 4.3. Injurious Compression and Treatment with PC and IL-10

After equilibration half of the cartilage explants were injured by subjection to an unconfined axial compression (injury) using an incubator-housed loading device, which has been previously described [21]. Controlled displacement ramp to 50% final strain was applied to each individual explant, using the original explant cutting thickness as starting point; ramp velocity was 2 mm/s (strain rate 200%/s). The non-porous platen was held for 10 s and then leveled back to the starting position (peak stress: 22.7 MPa, 95% CI 18.9–24.1). Afterwards, cartilage explants were transferred into chondropermissive medium and split into four different treatment groups: non-supplemented, additional IL-10 [100 pg/mL] (Kingfisher Biotech, Saint-Paul, MN, USA), additional PC (10% *v*/*v*) and PC (10% *v*/*v*) supplemented with exogenous IL-10 [100 pg/mL].

CS samples were treated following equilibration with the same four treatment conditions. Both, cartilage explants and CS, were cultivated in 250 µL medium at 37 °C in an atmosphere of 5% CO_2_. Medium change was twice a week.

### 4.4. DNA Quantification and Glycosaminoglycan Synthesis

Cell culture supernatant after 3 and 42 days was collected and stored frozen (−20 °C). After 42 days in culture, explants and CS samples were collected and snap frozen in liquid nitrogen following pulverization. Afterwards 1 mL deionized water was added and incubated for 30 min at room temperature. Sulphated glycosaminoglycan (sGAG) content and the culture supernatant was detected with modified 1,9-dimethylmethylene blue (DMMB) assay (Sigma-Aldrich, Darmstadt, Germany) as previously described [19]. The DNA content of the samples was quantified using a DNA Quantification Kit (Promega, Mannheim, Germany) according to manufacturer’s protocol. Total sGAG content and sGAG release was normalized to DNA content of the corresponding sample. Cumulative sGAG release into the cell culture supernatant was measured after each medium change over 42 days and then normalized to the DNA content of the corresponding sample after 42 days.

### 4.5. Relative Quantification PCR (qPCR)

Expression of mRNA was analyzed after 3 days of culture. Human cartilage explants and CS grafts were snap frozen in liquid nitrogen and pulverized. Afterwards cells were lysed by addition of 300 μL RLT-buffer (Qiagen, Hilden, Germany) with 1% 2-mercaptoethanol (Qiagen, Hilden, Germany). Cell lysate was separated from pulverized scaffolds debris by centrifugation using a QiaShredder (Qiagen, Hilden, Germany) column. Total mRNA content was extracted using the RNeasy Mini Kit with additional DNase I digestion according to the manufacturer’s instructions (Qiagen, Hilden, Germany). Complementary DNA (cDNA) was obtained by reverse transcription (RT) using Revert Aid H Minus Reverse Transcriptase (Thermofisher Scientific). Quantitative real-time PCR (qPCR) was performed using QuantiTect SYBR^®^ Green RT-qPCR Kit (Qiagen, Hilden, Germany) according to manufactures instructions with a 7500 Fast Real-Time PCR System (Applied Biosystems, Darmstadt, Germany). Human primers (Table 3) for aggrecan (ACAN), type 2 collagen (COL2A1), transcription factor SOX-9 (SOX9), type 1 collagen (COL1A1), type 10 collagen (COL10A1) and glyceraldehyde 3-phosphate dehydrogenase (GAPDH) (all from Sigma-Aldrich, Darmstadt, Germany) were used at a concentration of 0.3 μM. Data analysis was performed using a comparative quantification (∆∆CT-method). Using this method n-fold mRNA expression for the gene of interest was calculated using GAPDH as reference gene, which was evenly expressed throughout all experimental groups, and uninjured, untreated cartilage explants and untreated CS group as calibrator, respectively.

### 4.6. Histology—Detection of Cell Death, Toluidine Blue Staining and Immunohistochemistry

After 4 and 42 days in culture, cartilage explants were fixed overnight in 4% paraformaldehyde in PBS at room temperature and embedded in Paraplast (Sigma-Aldrich, Darmstadt, Germany). Analysis after 42 was chosen based on the authors experience to detect histological changes due to degradation in human cartilage. CS samples were fixed using the same method after 28 days. Serial sections of 7 µm were cut sagittally through the entire thickness of the samples (top-to-bottom) and then placed on glass slides. Cartilage explants were stained with Mayer’s haematoxylin to visualize cell apoptosis via detection of nuclear blebbing [36]. Images were taken using a Zeiss Axiophot microscope (Zeiss, Wetzlar, Germany) and the number of apoptotic cells was quantified [in %] in relation to the total number of cells per optical field by a blinded investigator. While margins of the sections (150 µm depth) were excluded, the value for each explant was calculated from three different areas. In addition, cartilage explants (after 42 days) and CS grafts (after 28 days) were stained with toluidine blue and immunostained for type 2 collagen (mouse anti-type-2-collagen antibody; CIIC1, DSHB, Iowa, USA; as described in [37] and type 1 collagen (mouse anti type-1-collagen antibody; CIIC1, DSHB, Iowa, USA; as described in [16].

### 4.7. Statistics

All data were tested for normality using the Kolmogorov-Smirnov test. Statistical analysis was performed using Graph Pad prism 8 program (Graph Pad Software Inc., San Diego, CA, USA). One-way-ANOVA analysis with Bonferroni‘s multiple comparisons was used to compare means among the independent experimental groups. Differences were considered significant if *p* ≤ 0.05, *p* ≤ 0.01, *p* ≤ 0.001 and *p* ≤ 0.0001. Quantitative data in the manuscript are presented as mean and standard deviation (SD).

## 5. Conclusions

PC has been demonstrated to mediate crucial chondroprotective mechanisms in mechanically injured cartilage making it a promising agent in controlling post-traumatic joint environment, especially in ACI treatment. However, additionally administering IL-10 may be necessary to improve targeted chondrogenic differentiation most effectively in post-injurious ACI.

## Figures and Tables

**Table 1:** Changes in transcription levels of chondrogenic markers and makers of de-differentiation in human cartilage explants.

	**COL2A1**	**Mean**	**±SD ^#^**	***p* ^†^**
a	+IL-10	1.24	0.65	** e, ** f
b	+PC	2.1	1.4	** e, * f
c	injury	1.53	0.9	** e, * f
d	injury + IL-10	3.74	2.19	ns
e	injury + PC	7.56	5.5	ns
f	injury + IL-10 + PC	7.44	3.91	ns
	**ACAN**	**Mean**	**±SD**	***p***
a	+IL-10	1.14	0.64	** e, * f
b	+PC	2.74	2	* e
c	injury	2.13	1.34	** e
d	injury + IL-10	4.44	2.68	ns
e	injury + PC	7.22	5	ns
f	injury + IL-10 + PC	5.88	2.87	ns
	**SOX9**	**Mean**	**±SD**	***p***
a	+IL-10	2.21	0.81	ns
b	+PC	5.88	5.79	** c
c	injury	0.63	0.37	ns
d	injury + IL-10	1.94	1.74	ns
e	injury + PC	4.24	2.8	ns
f	injury + IL-10 + PC	2.33	0.97	ns
	**COL1A1**	**Mean**	**±SD**	***p***
a	+IL-10	1.02	1.3	* c
b	+PC	1.88	1.59	ns
c	injury	8.89	9.07	ns
d	injury + IL-10	4.19	4.42	ns
e	injury + PC	4.53	5.53	ns
f	injury + IL-10 + PC	2.03	2.01	ns
	**COL10A1**	**Mean**	**±SD**	***p***
a	+IL-10	0.61	0.51	**** c
b	+PC	0.9	0.58	**** c
c	injury	2.84	1.38	ns
d	injury + IL-10	0.59	0.43	**** c
e	injury + PC	0.67	0.62	**** c
f	injury + IL-10 + PC	0.25	0.36	**** c
	**COL2/1 ratio**	**Mean**	**±SD**	***p***
a	+IL-10	1.98	2.27	ns
b	+PC	1.87	1.48	ns
c	injury	0.78	0.82	ns
d	injury + IL-10	3.9	4.05	ns
e	injury + PC	2.35	2.71	ns
f	injury + IL-10 + PC	2.95	2.34	ns

^#^ Standard deviation. ^†,^* *p* < 0.05, ** *p* < 0.01 and **** *p* < 0.0001, ns = not significant to any of the experimental groups.

**Table 2:** Changes in transcription levels of chondrogenic markers and makers of de-differentiation in autologous chondrocyte implantation (ACI) graft remains.

	**COL2A1**	**Mean**	**±SD ^#^**	***p* ^†^**
a	+IL-10	0.82	0.24	** c
b	+PC	1.09	0.16	* c
c	+IL-10 + PC	1.66	0.29	ns
	**ACAN**	**Mean**	**±SD**	***p***
a	+IL-10	2.8	0.66	ns
b	+PC	2.78	1.49	ns
c	+IL-10 + PC	2.41	1.34	ns
	**COL1A1**	**Mean**	**±SD**	***p***
a	+IL-10	0.81	0.21	ns
b	+PC	1.04	0.53	** c
c	+IL-10 + PC	0.31	0.1	ns
	**COL10A1**	**Mean**	**±SD**	***p***
a	+IL-10	1.6	0.66	ns
b	+PC	2.12	1.49	ns
c	+IL-10 + PC	1.04	1.34	ns
	**COL2/1 ratio**	**Mean**	**±SD**	***p***
a	+IL-10	0.94	0.12	** c
b	+PC	1.3	0.8	** c
c	+IL-10 + PC	5.91	1.77	ns

^#^ Standard deviation. ^†,^* *p* < 0.05, ** *p* < 0.01, ns = not statistically significant to any of the experimental groups.

**Table 3:** Primer sequences.

Human Target	Sequence (5′–3′) Sense	Sequence (5′–3′) Antisense
ACAN	GAGGCCAGCAGAGAAGATTCTG	GACGCCTCGCCTTCTTGAA
COL2A1	CAACACTGCCAACGTCCAGAT	CTGCTTCGTCCAGATAGGCAAT
SOX9	CTCGGAGACTTCTGAACGAGAG	CGTTCTTCACCGACTTCCTCC
COL1A1 COL10A1	AATTCCAAGGCCAAGAAGCATG CCCTTTTTGCTGCTAGTATCCTTGA	GGTAGCCATTTCCTTGGTGGTT AACTGTGTCTTGGTGTTGGGTAGTG
GAPDH	GCCTCAAGATCATCAGCAATGC	TGGTCATGAGTCCTTCCACGAT

## Data Availability

Data can be requested from the corresponding author.
